# Customer trends in take-away purchasing: Geospatial patterns of online food delivery platform usage in UK output areas

**DOI:** 10.23889/ijpds.v8i3.2275

**Published:** 2023-09-11

**Authors:** Tamara Garcia del Toro, Francesca Pontin, Rachel Oldroyd, Stephen Clark, Nik Lomax

**Affiliations:** 1 University of Leeds

## Introduction & Background

Current research in people’s diet habits has been very focused in the food environment: the different contexts in which people engage with the food system. Originally, this concept referred to the physical presence of food in a person’s surroundings, which affects their ability to access different foods.

The food environment has been transformed in the past decade, with the development of new services such as online grocery and take away delivery services. Alongside a shift towards more out-of-home-food consumption and the unique current historical context (COVID-19 pandemic and the cost-of-living crisis).

Previous work carried out by Keeble et al (2021) has looked at association between area outlet availability, online delivery platform usage and area deprivation, showing a positive association between number of food outlets available only, online delivery service usage, and area deprivation using scraped and self-reported data. However, no work to date has been able to look at transaction record to validate these results and better understand the demographic characteristics of ordering populations.

## Objectives & Approach

To better understand consumer habits around takeaway purchasing, and how the growth of online food delivery services has shaped new behaviours, we have partnered with a large online takeaway delivery platform to use their transaction data in order to shed light on how changing customer habits are shaping the food environment.

Over 5 million rows of transaction data for online food purchasing were provided by the data partner, a large online food delivery service. The data included anonymised customer reference id, location and order information, as well as food outlet details. Data were accessed through the retailer’s own secure platforms. Data analysis was carried out in two phases: an exploration of the locational characteristics of these classifications and distribution across UK geography, and exploration of fitted linear regression models to explain median basket price per output area.

Geodemographic data was sourced from the 2011 and 2021 census at the Output Area Level (approximately 125 households) and retailer data were matched using postcode information.

Model performance was estimated using the adjusted R2 coefficient and p-value for statistical significance, and further diagnostics tests included different residuals plots.

## Relevance to Digital Footprints

Self-reported nutrition data has been notoriously difficult to work with due to unreliability of memory and stigma.

Understanding people's eating habits is important if we are to understand how nutrition impacts health outcomes, how people interact with the food environment, which interventions are working, and to identify vulnerable populations.

Much research using digital footprints data to carry out nutrition research has focused around supermarket transaction data, which is limited as it does not clarify how the food is consumed if at all.

The current rise of the online food delivery market is helping create a shape the digital food environment, which is affecting and displacing the physical food environment. The data generated by users of online food delivery platforms is allowing us to closely understand how people are interacting with the food systems by adding a higher level of detail than self-reported data could.

## Results

We found that OAs with higher percentages of affluent homes had lower order frequency, with car ownership playing the biggest role.

We carried out a geospatial analysis of basket price, order frequency and percentage of deprived homes in Leeds and found that higher percentage of deprived homes mapped well over areas with high order frequency and low basket price.

## Conclusions & Implications

We found that demographic markers of affluence were highly associated with a higher median basket price and lower order frequency, and these were significantly able to predict median basket price per OA using fitted linear regression models.

The best predictors of median basket spend were car ownership and output area classification supergroup. We believe there to be a complex interplay of deprivation and access factors which are best captured by these measures.

We found that more deprived population have a higher number of orders with lower basket prices. We have seen that higher order frequency is associated with a higher number of orders to restaurants which cuisine type is defined as burgers. Cuisine type preferences might be able to explain median basket price difference between affluent and deprived populations. Further work should look to see whether availability of different cuisine types differs by area to understand how access is driving cuisine preference.

